# Supplementary material for: Iterative improvement in the automatic modular design of robot swarms
Source: PeerJ Comput Sci. 2020 Dec 7;6:e322. doi: 10.7717/peerj-cs.322 (PMC7924708; doi:10.7717/peerj-cs.322)
Supplement: Supplemental Information 3 [file peerj-cs-06-322-s003.zip › argos3/doc/api/standalone/a00607_source.html]

ARGoS: plugins/simulator/physics\_engines/dynamics2d/dynamics2d\_engine.h Source File


- Main Page
- Related Pages
- Namespaces
- Classes
- Files

- File List
- File Members

# plugins/simulator/physics\_engines/dynamics2d/dynamics2d\_engine.h

Go to the documentation of this file.

```
00001 
00007 #ifndef DYNAMICS2D_ENGINE_H
00008 #define DYNAMICS2D_ENGINE_H
00009 
00010 namespace argos {
00011    class CDynamics2DEngine;
00012    class CDynamics2DModel;
00013    class CGripperEquippedEntity;
00014    class CEmbodiedEntity;
00015 }
00016 
00017 #include <argos3/core/simulator/entity/controllable_entity.h>
00018 #include <argos3/core/simulator/physics_engine/physics_engine.h>
00019 #include <argos3/plugins/simulator/physics_engines/dynamics2d/chipmunk-physics/include/chipmunk.h>
00020 
00021 namespace argos {
00022 
00023    /****************************************/
00024    /****************************************/
00025 
00026    struct SDynamics2DEngineGripperData {
00027       CDynamics2DEngine& Engine;
00028       CGripperEquippedEntity& GripperEntity;
00029       cpVect GripperAnchor;
00030       cpConstraint* GripConstraint;
00031 
00032       SDynamics2DEngineGripperData(CDynamics2DEngine& c_engine,
00033                                    CGripperEquippedEntity& c_entity,
00034                                    cpVect t_anchor);
00035       ~SDynamics2DEngineGripperData();
00036       void ClearConstraints();
00037    };
00038 
00039    /****************************************/
00040    /****************************************/
00041 
00042    class CDynamics2DEngine : public CPhysicsEngine {
00043 
00044    public:
00045 
00046       enum EShapeType {
00047          SHAPE_NORMAL = 0,
00048          SHAPE_GRIPPABLE,
00049          SHAPE_GRIPPER
00050       };
00051 
00052       enum ELayerType {
00053          LAYER_FROZEN = 0,
00054          LAYER_NORMAL = CP_ALL_LAYERS
00055       };
00056 
00057       CDynamics2DEngine();
00058 
00059       virtual ~CDynamics2DEngine() {}
00060 
00061       virtual void Init(TConfigurationNode& t_tree);
00062       virtual void Reset();
00063       virtual void Update();
00064       virtual void Destroy();
00065 
00066       virtual size_t GetNumPhysicsModels();
00067       virtual bool AddEntity(CEntity& c_entity);
00068       virtual bool RemoveEntity(CEntity& c_entity);
00069 
00070       virtual void CheckIntersectionWithRay(TEmbodiedEntityIntersectionData& t_data,
00071                                             const CRay3& c_ray) const;
00072 
00073       inline cpSpace* GetPhysicsSpace() {
00074          return m_ptSpace;
00075       }
00076 
00077       inline const cpSpace* GetPhysicsSpace() const {
00078          return m_ptSpace;
00079       }
00080 
00081       inline cpBody* GetGroundBody() {
00082          return m_ptGroundBody;
00083       }
00084 
00085       inline Real GetElevation() const {
00086          return m_fElevation;
00087       }
00088 
00089       inline Real GetDamping() const {
00090          return cpSpaceGetDamping(m_ptSpace);
00091       }
00092 
00093       inline void SetDamping(Real f_damping) {
00094          cpSpaceSetDamping(m_ptSpace, f_damping);
00095       }
00096 
00097       inline CVector2 GetGravity() const {
00098          return CVector2(m_ptSpace->gravity.x, m_ptSpace->gravity.y);
00099       }
00100 
00101       inline void SetGravity(const CVector2& c_gravity) {
00102          m_ptSpace->gravity = cpv(c_gravity.GetX(), c_gravity.GetY());
00103       }
00104 
00105       void PositionPhysicsToSpace(CVector3& c_new_pos,
00106                                   const CVector3& c_original_pos,
00107                                   const cpBody* pt_body);
00108 
00109       void OrientationPhysicsToSpace(CQuaternion& c_new_orient,
00110                                      cpBody* pt_body);
00111       
00112       void AddPhysicsModel(const std::string& str_id,
00113                             CDynamics2DModel& c_model);
00114       void RemovePhysicsModel(const std::string& str_id);
00115 
00116    private:
00117 
00118       cpFloat m_fStaticHashCellSize;
00119       cpFloat m_fActiveHashCellSize;
00120       SInt32 m_nStaticHashCells;
00121       SInt32 m_nActiveHashCells;
00122       cpSpace* m_ptSpace;
00123       cpBody* m_ptGroundBody;
00124       Real m_fElevation;
00125 
00126       CControllableEntity::TMap m_tControllableEntities;
00127       std::map<std::string, CDynamics2DModel*> m_tPhysicsModels;
00128 
00129    };
00130 
00131    /****************************************/
00132    /****************************************/
00133 
00134    template <typename ACTION>
00135    class CDynamics2DOperation : public CEntityOperation<ACTION, CDynamics2DEngine, SOperationOutcome> {
00136    public:
00137       virtual ~CDynamics2DOperation() {}
00138    };
00139 
00140    class CDynamics2DOperationAddEntity : public CDynamics2DOperation<CDynamics2DOperationAddEntity> {
00141    public:
00142       virtual ~CDynamics2DOperationAddEntity() {}
00143    };
00144 
00145    class CDynamics2DOperationRemoveEntity : public CDynamics2DOperation<CDynamics2DOperationRemoveEntity> {
00146    public:
00147       virtual ~CDynamics2DOperationRemoveEntity() {}
00148    };
00149 
00150 #define REGISTER_DYNAMICS2D_OPERATION(ACTION, OPERATION, ENTITY)        \
00151    REGISTER_ENTITY_OPERATION(ACTION, CDynamics2DEngine, OPERATION, SOperationOutcome, ENTITY);
00152 
00153 #define REGISTER_STANDARD_DYNAMICS2D_OPERATION_ADD_ENTITY(SPACE_ENTITY, DYN2D_MODEL) \
00154    class CDynamics2DOperationAdd ## SPACE_ENTITY : public CDynamics2DOperationAddEntity { \
00155    public:                                                              \
00156    CDynamics2DOperationAdd ## SPACE_ENTITY() {}                         \
00157    virtual ~CDynamics2DOperationAdd ## SPACE_ENTITY() {}                \
00158    SOperationOutcome ApplyTo(CDynamics2DEngine& c_engine,               \
00159                 SPACE_ENTITY& c_entity) {                               \
00160       DYN2D_MODEL* pcPhysModel = new DYN2D_MODEL(c_engine,              \
00161                                                  c_entity);             \
00162       c_engine.AddPhysicsModel(c_entity.GetId(),                        \
00163                                *pcPhysModel);                           \
00164       c_entity.                                                         \
00165          GetComponent<CEmbodiedEntity>("body").                         \
00166          AddPhysicsModel(c_engine.GetId(), *pcPhysModel);               \
00167       return SOperationOutcome(true);                                   \
00168    }                                                                    \
00169    };                                                                   \
00170    REGISTER_DYNAMICS2D_OPERATION(CDynamics2DOperationAddEntity,         \
00171                                  CDynamics2DOperationAdd ## SPACE_ENTITY, \
00172                                  SPACE_ENTITY);
00173    
00174 #define REGISTER_STANDARD_DYNAMICS2D_OPERATION_REMOVE_ENTITY(SPACE_ENTITY) \
00175    class CDynamics2DOperationRemove ## SPACE_ENTITY : public CDynamics2DOperationRemoveEntity { \
00176    public:                                                              \
00177    CDynamics2DOperationRemove ## SPACE_ENTITY() {}                      \
00178    virtual ~CDynamics2DOperationRemove ## SPACE_ENTITY() {}             \
00179    SOperationOutcome ApplyTo(CDynamics2DEngine& c_engine,               \
00180                 SPACE_ENTITY& c_entity) {                               \
00181       c_engine.RemovePhysicsModel(c_entity.GetId());                    \
00182       c_entity.                                                         \
00183          GetComponent<CEmbodiedEntity>("body").                         \
00184          RemovePhysicsModel(c_engine.GetId());                          \
00185       return SOperationOutcome(true);                                   \
00186    }                                                                    \
00187    };                                                                   \
00188    REGISTER_DYNAMICS2D_OPERATION(CDynamics2DOperationRemoveEntity,      \
00189                                  CDynamics2DOperationRemove ## SPACE_ENTITY, \
00190                                  SPACE_ENTITY);
00191    
00192 #define REGISTER_STANDARD_DYNAMICS2D_OPERATIONS_ON_ENTITY(SPACE_ENTITY, DYN2D_ENTITY) \
00193    REGISTER_STANDARD_DYNAMICS2D_OPERATION_ADD_ENTITY(SPACE_ENTITY, DYN2D_ENTITY) \
00194    REGISTER_STANDARD_DYNAMICS2D_OPERATION_REMOVE_ENTITY(SPACE_ENTITY)
00195 
00196    /****************************************/
00197    /****************************************/
00198 
00199 }
00200 
00201 #endif
```

---

Generated on 10 Jul 2018 for ARGoS by 
 1.6.1
